# Catheter‐based induction of renal ischemia/reperfusion in swine: description of an experimental model

**DOI:** 10.14814/phy2.12150

**Published:** 2014-09-28

**Authors:** Pamella A. Malagrino, Gabriela Venturini, Patrícia S. Yogi, Rafael Dariolli, Kallyandra Padilha, Bianca Kiers, Tamiris C. Gois, Joaquim M. da Motta‐Leal‐Filho, Celso K. Takimura, Adriana C. C. Girardi, Francisco C. Carnevale, Ana C. M. Zeri, Denise M. A. C. Malheiros, José E. Krieger, Alexandre C. Pereira

**Affiliations:** 1 Laboratory of Genetics and Molecular Cardiology, Heart Institute, University of São Paulo Medical School, São Paulo, SP, Brazil; 2 Interventional Radiology Unit, Department of Radiology, Heart Institute, University of São Paulo Medical School, São Paulo, SP, Brazil; 3 Interventional Radiology Unit, Radiology Institute, Hospital das Clínicas, University of São Paulo Medical School, São Paulo, SP, Brazil; 4 Biosciences National Laboratory, LNBio, Campinas, SP, Brazil; 5 Department of Pathology, University of São Paulo Medical School, São Paulo, SP, Brazil

**Keywords:** Acute kidney injury, acute tubular necrosis, balloon‐catheter, ischemia, renal artery occlusion, swine model

## Abstract

Several techniques to induce renal ischemia have been proposed: clamp, PVA particles, and catheter‐balloon. We report the development of a controlled, *single‐insult* model of unilateral renal ischemia/reperfusion (I/R) without contralateral nephrectomy, using a suitable model, the pig. This is a balloon‐catheter‐based model using a percutaneous, interventional radiology procedure. One angioplasty balloon‐catheter was placed into the right renal artery and inflated for 120 min and reperfusion over 24 h. Serial serums were sampled from the inferior vena cava and urine was directly sampled from the bladder throughout the experiment, and both kidneys were excised after 24 h of reperfusion. Analyses of renal structure and function were performed by hematoxylin–eosin/periodic Acid‐Schiff, serum creatinine (SCr), blood urea nitrogen (BUN), fractional excretion of ions, and glucose, SDS‐PAGE analysis of urinary proteins, and serum neutrophil gelatinase‐associated lipocalin (NGAL). Total nitrated protein was quantified to characterize oxidative stress. Acute tubular necrosis (ATN) was identified in every animal, but only two animals showed levels of SCr above 150% of baseline values. As expected, I/R increased SCr and BUN. Fractional sodium, potassium, chloride, and bicarbonate excretion were modulated during ischemia. Serum‐nitrated proteins and NGAL had two profiles: decreased with ischemia and increased after reperfusion. This decline was associated with increased protein excretion during ischemia and early reperfusion. Altogether, these data show that the renal I/R model can be performed by percutaneous approach in the swine model. This is a suitable translational model to study new early renal ischemic biomarkers and pathophysiological mechanisms in renal ischemia.

## Abstract (precis)

e12150

We report the development of a controlled, single‐insult model of unilateral renal ischemia/reperfusion (I/R) without contralateral nephrectomy, using a suitable model, the pig. This model can be useful for translational studies, including development of new diagnosis biomarkers and drugs studies. Furthermore, this kind of data is of paramount importance to better understand the pathophysiological mechanisms of renal disease.

## Introduction

Ischemia is a common cause of acute kidney injury (Thadhani et al. 1996) (AKI) resulting from a generalized or localized decrease in oxygen and nutrient support to the tissue. These processes lead to increased immune responses (Bonventre and Zuk 2004), structural/functional kidney injury (Le Dorze et al. 2009), and reactive oxygen species (ROS). During reperfusion, the migration of ROS into the organ contributes to cellular damage and may lead to acute tubular necrosis (ATN) and organ dysfunction (Li and Jackson 2002). Ischemic acute kidney injury is often associated with multiple organ failure and unsuccessful renal transplantation. In addition, medications and many pathological states, such as renal artery thrombosis and stenosis, vasculitis, or malignant hypertension, can contribute to generalized or localized ischemia (Bonventre and Yang 2011). Patients after cardiac surgery and critically ill patients are the most affected by renal ischemia. More than 36% of critically ill patients develop AKI, which significantly increases the mortality rate (Bagshaw et al. 2008).

According to the “Acute Kidney Injury Network” (AKIN), AKI is defined as an abrupt decrease in kidney function, and the diagnosis is based on the concentration of serum creatinine or urine output. Stages of this disease are classified by AKIN criteria (Mehta et al. 2007). Currently, it is well established that serum creatinine is a late biomarker (Shemesh et al. 1985), and its level varies with age, sex, muscle metabolism, and hydration (Nguyen and Devarajan 2008). Other biomarkers, such as neutrophil gelatinase‐associated lipocalin (NGAL), have been tested in clinical practice (Boldt et al. 2009; Du et al. 2011) with promising results (Mishra et al. 2005).

To investigate early biomarkers and the mechanisms responsible for renal ischemia/reperfusion (I/R), suitable animal models are paramount. Several studies have reported different animal models of renal I/R, such as mice (Matthijsen et al. 2007; Susa et al. 2009), rats (Damianovich et al. 2006; Bhalodia et al. 2009), rabbits (Formiga et al. 2011; Zhen‐Qiang et al. 2012), and dogs (Tsuji et al. 1993; Yatsu et al. 1998). However, pigs may be a more suitable model to study human renal I/R consequences (Lieberthal and Nigam 2000; Simmons et al. 2008) due to their characteristics, such as renal embryology, multilobular anatomy, multipapillary architecture, lymphatic pattern, ability to concentrate urine, tolerance to ischemia, immune response, and homology (Lieberthal and Nigam 2000; Lunney 2007 and Piriou‐guzylack and Salmon 2008). Despite the fact that pigs have been used extensively as preclinical models of cardiovascular (Ho et al. 2009), renal (Jochmans et al. 2011), and intestinal diseases (Korth et al. 2003), sparse data are available on swine renal I/R models.

Herein, we describe a suitable, controlled, *single‐insult* model of unilateral renal I/R without contralateral nephrectomy in pigs. This is a balloon‐catheter‐based model with a minimally invasive interventional radiology procedure. The model seems to be especially suitable for the identification and characterization of biomarkers of kidney injury and the assessment of pathophysiological responses during ischemia and reperfusion, common in cardiac surgery (systemic I/R) and renal transplantation (injury site I/R).

## Methods

Experiments were conducted according to the protocol approved by the institutional ethics committee (CAPPesq Protocol 179/11) and performed according to the Guiding Principles for Research Involving Animals. Five female pigs (*Sus scrofa domesticus*, MS60 EMBRAPA lineage – weight, 15–20 kg and 8–12 weeks old) were obtained from a local commercial swine farm (Granja RG, Suzano‐SP, Brazil). This age range was chosen due to the absence of sexual maturity (4–6 months) (Smith and Swindle 2006), avoiding hormone interferences. Only animals with no signs of diseases were used. Before the experiment, animals were acclimatized at least 48 h in an experimental facility. Food was withdrawn 12 h before ischemia induction, and released ad libitum after surgery.

Animals were sedated with a mixture of ketamine chloridrate (8 mg/kg, Dopalen^®^, Vetbrands, São Paulo, Brazil) and midazolam chloridrate through intramuscular injection (0.5 mg/kg; Dormonid^®^, Roche, Rio de Janeiro, Brazil). After 10–20 min, anesthesia was induced with sodium thiopental (12.5 mg/kg; Thiopentax^®^, Cristalia, São Paulo, Brazil) through a cannula introduced into a superficial ear vein, and then orotracheal intubation (7‐ to 7.5‐mm tube, Chilecom, Guangdong, China) was performed. Anesthesia was maintained with isoflurane (1.5 to 2.5% – Isothane^®^ Baxter, Guayama, Puerto Rico) in 100% oxygen in anesthetic equipment (Origami Ergo System, Takaoka).

### Percutaneous induction of acute renal ischemia

Anesthetized pigs were fixed in a supine position. First, a Foley urinary catheter was surgically placed into the bladder by dissection to collect urinary samples.

After inguinal asepsis/antisepsis, a 6‐*French* (F) vascular sheath (Merit Medical's, Systems; South Jordan, UT, USA) was introduced by dissection of the right common femoral vein followed by cannulation of the inferior vena cava with a straight catheter placed above the renal veins to collect venous blood samples. Administration of 10,000 units of heparin (Hemofol^®^, Cristalia, São Paulo, Brazil) was performed. Another 6F vascular sheath (Merit Medical Systems) was introduced by dissection of the right common femoral artery.

Selective catheterization of the right renal artery was performed using a 6F multipurpose guide‐catheter and a 0.035″ guide‐wire followed by renal artery injection of iodinated contrast medium **(**Pielograf^®^ 76% – Bracco Diagnostics Inc, Monroe Township, NJ) and baseline angiography was acquired. Fluoroscopic equipment used Philips BV pulsera. To perform the occlusion of the right renal artery, a guide‐wire 0.014″ (Merit Medical Systems, South Jordan, UT) was introduced followed by a 6F balloon‐catheter 5 × 20 mm; (Cordis M3 PTA dilatation catheter, Bridgewater, NJ). Balloon size was selected in accordance with the main renal artery diameter and length, and inflated in the proximal portion of the right renal artery, totally occluding the blood flow to the right kidney for up to 120 min. Two hours after occlusion, the balloon was deflated and carefully removed. A new angiography was performed to show right renal artery patency and renal reperfusion. The guide‐catheter, guide‐wire 0.014″, and 6F sheath were removed and the common femoral artery was sutured. The animals were monitored and maintained on mechanical ventilation until the resumption of normal vital functions (at least 3 h). After the end of anesthesia effect, the animals were removed and accommodated in a temperature‐controlled room with water and food ad libitum. Sodium dipyrone (50 mg/mL) was injected via a peripheral vein every 8 h. During all procedures, a solution of sodium chloride 0.9% was infused 170 mL/h.

### Serial serum and urinary sampling

**Figure 1.**
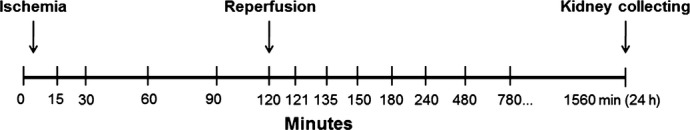
Schema of blood and urine sampling. Serial serum and urine were sampled before ischemia (0), during ischemia (1, 15, 30, 60, 90, and 120 min after occlusion), and after balloon deflation, in reperfusion (121, 135, 150, 180 min and then at intervals of 1 h for 24 h). After 24 h of reperfusion, kidneys were collected for analysis.

Animals underwent serial sampling of venous blood and urine for up to 24 h. Serum samplings were performed by the cannula introduced into the inferior vena cava above the renal veins. Five milliliters of blood was collected in sterile syringes and placed in tubes (Serum Clot Activator tubes, VACUETTE^®^, Greiner Bio‐One, Monroe, NC) and 50 mL of urine was collected. Blood and urinary samples were centrifuged at 2950 × g for 10 min. Serum was aliquoted in tubes with proteases and phosphatase inhibitor cocktails (P8340, P5726, P0044, Sigma‐Aldrich, St. Louis, MO) and then stored at −80°C until further analysis, and urine was only aliquoted and stored at −20°C. Serial serum and urine samplings were performed before ischemia (0), during ischemia (1, 15, 30, 60, 90, and 120 min after occlusion), and after balloon deflation (121, 135, 150, 180 min, and then at intervals of 1 h for 24 h) (Fig. 1). After 24 h of serial sampling, the animals were sedated with a mixture of ketamine chloridrate (8 mg/kg, Vetbrands) and midazolam (0.5 mg/kg; Roche), followed by a high dose of sodium thiopental (30 mg/kg; Cristalia), and then the animals were killed with a high dose of potassium chloride 0.9% (20–30 mL).

### Histopathological characterization of renal injury

For histopathologic analysis, ischemic and nonischemic tissues fixed in paraformaldehyde were paraffin embedded, sectioned at 5 μm thickness, and stained with hematoxylin–eosin (HE) and periodic acid‐Schiff (PAS). The tissue was evaluated by one expert pathologist using light microscopy who was blind to the study protocol. The renal cortex sections were examined for loss of proximal tubule brush border, patchy loss of tubular cells, focal areas of proximal tubular dilation, and distal tubular casts in ischemic and contralateral kidneys. The fields for each kidney slide were examined semiquantitatively and assigned for severity of change intervals of 10–25%, 26–50%, and 51–100% of area with ATN.

### Neutrophil gelatinase‐associated lipocalin (NGAL)

Neutrophil gelatinase‐associated lipocalin levels in serum were determined by a commercially available ELISA assay according to the manufacturer's recommendations (Bioporto Diagnostics, Gentofte, Denmark).

### Nitrated proteins

Serum total nitrated proteins were measured using a nitrotyrosine sandwich ELISA kit (Eastbiopharm – China) following manufacturer's instructions. Urine was concentrated (cut off 3 KDa; Amicon Millipore, Billerica, MA) followed by dot‐blot analysis to assess total nitrated proteins. Nitrocellulose membrane was loaded with 2 μL of urine, BSA (negative control), and nitrated BSA (positive control). The membrane was incubated against polyclonal nitrotyrosine antibody (1:1000) (Millipore #06‐284) for 2 h at room temperature. Nitrated proteins were detected using goat anti‐rabbit conjugated to alkaline phosphatase and NBT/BCIP (Ref. 00‐2209 – Invitrogen, Camarillo, CA, USA). ImageQuant TL (GE) was used for quantification. All samples were normalized by protein amount quantified using Bradford methods. The final results were obtained by dividing the pixels assessed in dot‐blot by total proteins and creatinine amount.

### Biochemical characterization of renal injury

Serum creatinine concentration was measured using the Jaffé method. The animals were classified as AKI according to the AKIN (Mehta et al. 2007), in which the diagnosis of AKI is defined by an increase in serum creatinine level above 150–200% from baseline.

Serum urea was measured by UV UREASI/GLDH kinetic. Urinary and serum sodium, potassium, chloride, urinary pH, and partial pressure of carbon dioxide (pCO_2_) in urine were measured on a Radiometer ABL800Flex (Radiometer Medical, Bronshoj, Demmark). Urinary bicarbonate concentration was calculated using the Handerson–Hasselbach equation. All these data were used to calculate the fractional excretion of ions. Urine protein excretion was quantified using a Sensiprot kit (Labtest, Minas Gerais, Brazil) and qualified by 10% SDS‐PAGE containing 10 μg of creatinine and 2 μg of bovine serum albumin (BSA) as a positive control, silver stained using a Proteosilver Plus kit (Sigma). Urinary glucose and creatinine were measured before ischemia (0), during ischemia (60 min), and post reperfusion (135, 360, 780, and 1080 min).

### Statistical analysis

Statistical analyses were performed with GraphPad Prism 5 (GraphPad Software Inc, San Diego, CA) software. Continuous variables among groups were tested for normality with the Kolmogorov–Smirnov test and analyzed by one‐way repeated measure ANOVA. Values are shown as mean ± standard error of the mean (SEM). A level of *P* < 0.05 was set as statistically significant.

## Results

### Procedure assessment

**Figure 2.**
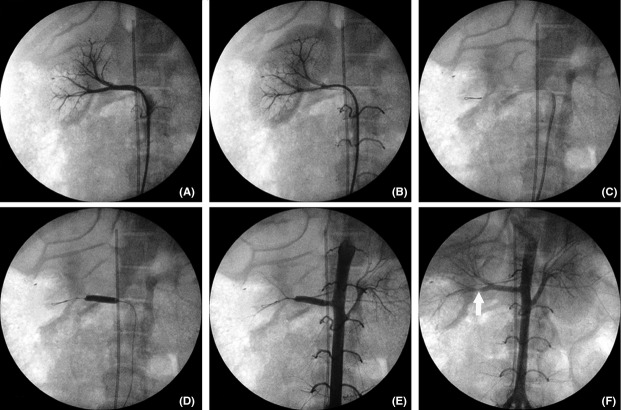
Angiographic representation of the right renal system. (A) Arterial phase of a selective digital subtraction angiogram of the right renal artery before ischemia showing normal vascular arrangement. (B) Late phase of same angiogram showing right kidney parenchyma. (C) A 0.014″ guide‐wire was placed into the right renal artery branch (D) A 5 × 20 mm balloon‐catheter was inflated in the proximal portion of right renal artery to generate kidney ischemia for 120 min. (E) Angiogram of the abdominal aorta showing the complete occlusion of the right renal artery, without kidney vascularization. (F) Final angiogram of the abdominal aorta showing right renal reperfusion with a mild spasm at the distal portion of the artery after balloon withdrawn (white arrow), but not impairing reperfusion. Note in all images a straight catheter placed into the inferior vena cava above the renal veins.

The average handling time of the procedure between the dissection of the common femoral artery and vein, cannulation of the inferior vena cava, localization of renal artery, and skin suture was constant (64 ± 9 min). Angiographic images were obtained before the renal artery occlusion (Fig. 2A and B), while the guide‐wire 0.014″ was placed in the proximal portion of the right renal artery (Fig. 2C) and when the balloon was inflated in the proximal portion completely occluding the vessel (Fig. 2D and E). After balloon deflation, the blood circulation into the right renal artery was recovered and no significant spasm or dissection areas were observed (Fig. 2F). Pigs were monitored during the entire procedure, and no significant alterations in cardiac frequency or oxygen saturation were observed (data not shown).

### Histopathological assessment

**Figure 3.**
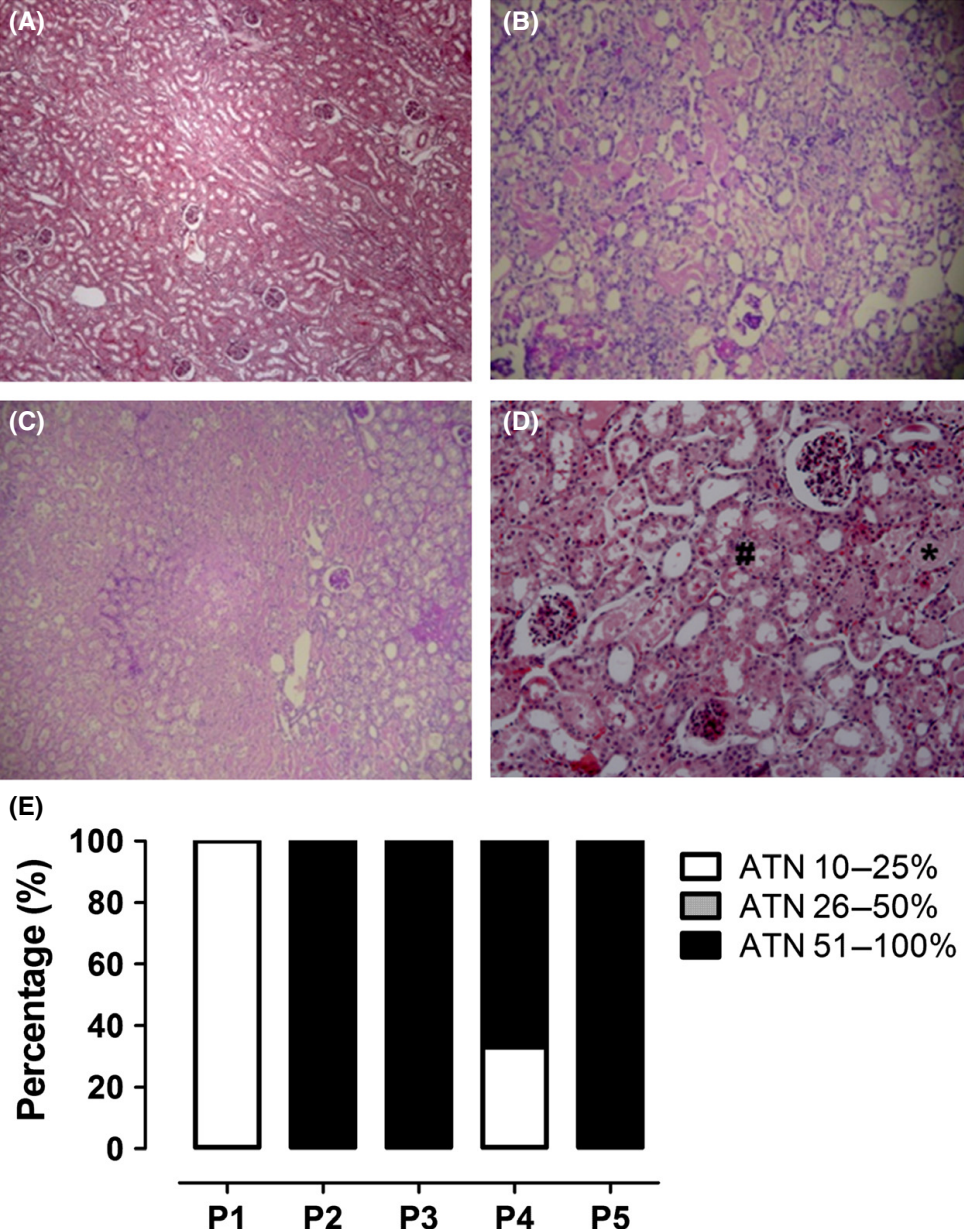
Histopathological assessment of the renal cortex. (A) Contralateral kidney, not exposed to ischemia and with no signs of ATN; (B) ATN in ischemic kidney; (C) White ischemia and ATN in more than 51% of tissue; Periodic Acid‐Schiff (PAS) stain, 50× magnifications; (D) Representative view of ATN. Note: appearance of tubular debris and casts (*) and loss of proximal tubular brush border (**#**). Hematoxylin–Eosin, 200× magnification. (E) Quantification of the percentage of ATN in ischemic kidneys of different animals (P1, P2, P3, P4, P5). Acute tubular necrosis (ATN).

In our model, the contralateral kidney did not show any structural changes (Fig. 3A). On the other hand, the ischemic kidney showed acute tubular necrosis (NTA) and/or areas with white and hemorrhagic necrosis (except in animal 1) (Fig. 3B and C). The ischemic kidney showed effacement and loss of proximal tubule brush border, patchy loss of tubular cells, focal areas of proximal tubular dilation, and distal tubular casts (Fig. 3D).

ATN was identified in 10–25% of the cortex of animal 1 (P1). In the other animals, ATN was quantified between 51 and 100% of the evaluated cortex (Fig. 3E).

### Biochemical assessment

#### Creatinine and BUN levels in serum

**Figure 4.**
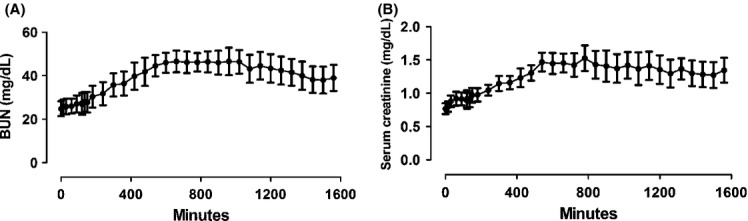
Serum creatinine and blood urea nitrogen (BUN) levels, before ischemia (0 min), during ischemia (1–120 min), and after reperfusion (121–1560 min). (A) Serum creatinine. (B) Blood urea nitrogen (BUN). Significant differences were observed in both metabolites versus preischemia assessment (0 min). Values are presented as mean ± SEM. Analysis for repeated‐measures (ANOVA) *P* < 0.05 (*n* = 5).

Renal function was assessed by measurement of serum creatinine and blood urea nitrogen (BUN). The levels of creatinine and BUN were significantly increased after occlusion (Fig. 4).

**Figure 5.**
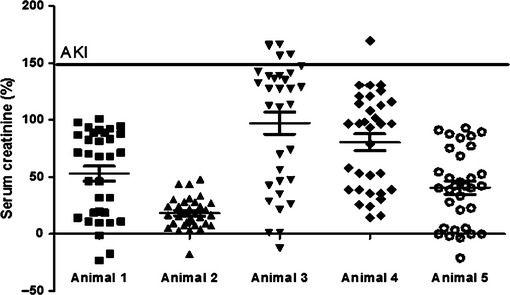
Percentile analyses of the increase in serum creatinine (regardless of sampling time). Note that animals 3 and 4 had serum levels above 150% therefore being classified as having AKI. Acute kidney injury (AKI).

However, based on AKIN (Mehta et al, 2007) criteria, animals could not be classified as having AKI, even 24 h after the procedure, contrary to the histological analysis (Fig. 3). Percentage analyses of the increase in creatinine were plotted in a chart of creatinine rate regardless of sampling time. Only animals 3 and 4 showed values above the minimum reference to AKI diagnostic (Fig. 5).

### Measurement of serum NGAL

**Figure 6.**
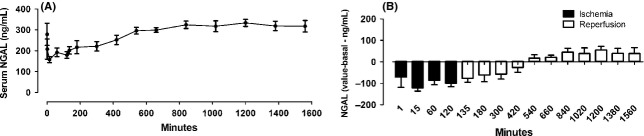
Neutrophil gelatinase‐associated lipocalin (NGAL) levels in blood serum along with ischemia and reperfusion. (A) Values of NGAL concentration, (B) Difference between the times of the experiment and baseline levels, emphasizing the increase of NGAL after reperfusion. This increase was significantly different when ischemia and reperfusion are compared. Values are presented as mean ± SEM. Analysis for repeated‐measures (ANOVA) *P* < 0.05 (*n* = 5).

Statistical decrease of serum NGAL was detected immediately after ischemia followed by an increase after reperfusion (Fig. 6A). Interestingly, only after 7 h of reperfusion were the levels of NGAL significantly higher than baseline levels (Fig. 6B).

### Oxidative stress (Nitrated proteins)

**Figure 7.**
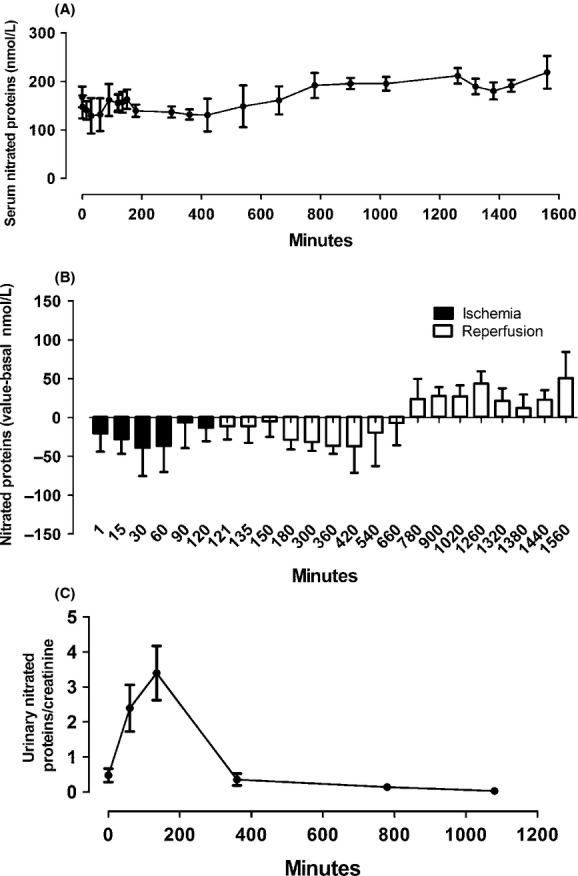
Serum and urinary‐nitrated proteins during ischemia and reperfusion. (A) Values of serum concentration of nitrated proteins. (B) Difference between the times of the experiment with baseline emphasizing the increase in nitrated protein after reperfusion. (C) Increase in oxidative stress in the kidney during ischemia shown in urinary‐nitrated protein levels (*n* = 4). All were significantly different. Values are presented as mean ± SEM. Analysis for repeated‐measures (ANOVA); *P* < 0.05.

The ischemia/reperfusion performed by the single‐insult, catheter‐based model in pigs lead to an increase in oxidative stress in the kidney. The systemic oxidative stress was assessed by the measurement of total nitrated protein in serum, showing a statistically significant increase after reperfusion (Fig. 7A and B). Interestingly, there was a significant increase in the excretion rate of nitrated proteins that recovered after reperfusion (Fig. 7C) in accordance with the serum.

### Urinary protein and glucose

**Figure 8.**
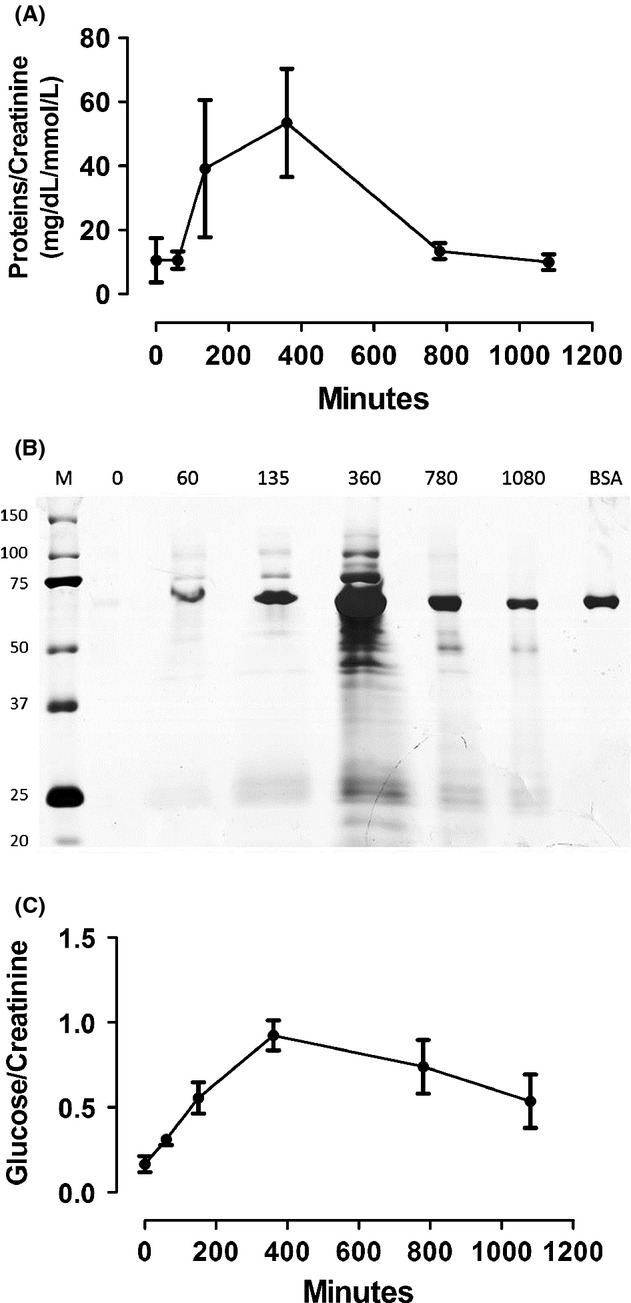
Urinary protein and glucose. (A) Urinary proteins/creatinine (*n* = 5) (B) Pattern urinary protein in electrophoresis. Albumin, 69 KDa. BSA, bovine serum albumin (2 μg). (C) Urinary glucose/creatinine (*n* = 4). All showed a significantly increase in glucose and protein excretion with ischemia. Values are presented as mean ± SEM. Analysis for repeated‐measures (ANOVA); *P* < 0.05.

After ischemia, we observed increased protein and glucose excretion into the urine (Fig. 8). Total protein excreted showed a significant increase peaking in 360 min after renal artery occlusion, decreasing to baseline levels in 24 h (Fig. 8A). Not only the amount of urinary proteins but also the urinary pattern of excreted proteins was changed after occlusion. Interestingly, proteins larger than albumin were observed after ischemia (Fig. 8B). Meanwhile, urinary excretion of glucose showed the same pattern of nitrated proteins, with a significant increase during ischemia and early reperfusion and peaking at 360 min after renal artery occlusion (Fig. 8C). Both levels tended to decrease at 24 h, but basal levels were not reestablished until the last assessed point.

### Fractional excretion of ions during renal I/R procedure

**Figure 9.**
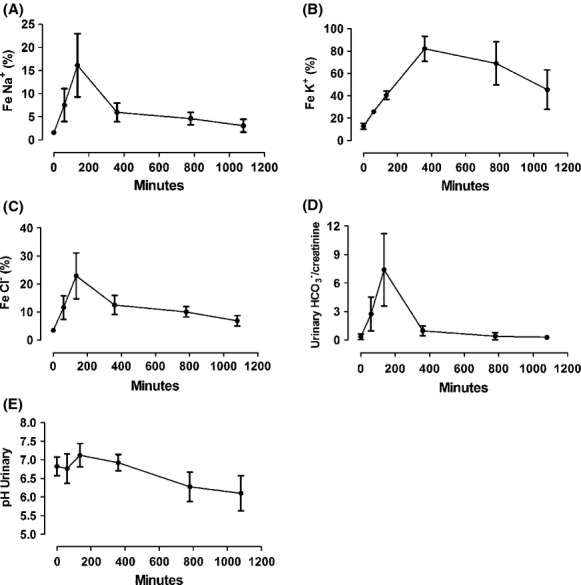
Urinary biochemical analysis during ischemia and reperfusion. (A) Fractional excretion of sodium (FeNa^+^). (B) Fractional excretion of potassium (FeK^+^). (C) Fractional excretion of chloride (FeCl^−^). (D) bicarbonate (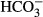)/creatinine excretion. (E) Urinary pH. All showed significant differences. Values are presented as mean ± SEM. Analysis for repeated‐measures (ANOVA); *P* < 0.05 (*n* = 5).

Ischemia caused an increase in the fractional excretion of ions. The fractional excretion of sodium significantly increased at the onset of reperfusion. Recovery of renal handling of sodium began 360 min after ischemia (Fig. 9A). Similarly, the fractional excretion of potassium increased significantly after ischemia but with a later peak (360 min after renal artery occlusion; Fig. 9B). As expected, the fractional excretion of chloride and the bicarbonate/creatinine ratio followed the same pattern of the sodium ion (Fig. 9C and D). As a consequence of the bicarbonate excretion, urinary pH also increased at the beginning of reperfusion (Fig. 9E).

## Discussion

In this study, we provide evidence that a catheter‐based renal I/R in pigs is a suitable, minimally invasive, model with precise, and reproducible occlusion of the renal artery. This method did not use nephrectomy and showed no mortality, which is often observed in conventional surgical models (Keller et al. 2008; Sabbagh et al. 2011).

Our model of ischemia and reperfusion aims to provide a well‐characterized animal model with strong acute responses to ischemic insult, similar to the human response. Our model did not aim to mimic a specific disease, but rather, provide common pathophysiological changes observed in various kidney diseases and transplant. Based on the others pigs' models of kidney ischemia (Weld et al. 2008b; Weld et al. 2008a; Matějková et al. 2013), we choose 120 min, an intermediary time among the models available in literature. The time of occlusion in pig models of ischemia is usually longer than observed in rodents. For example, pig models of heart ischemia showed three times longer occlusion time than rodent models. This occurs because the pig hearts show a basal energy turnover rate approximately three times lower than rat hearts (Kupriyanov et al. 1995). Renal I/R large animal models are required for new biomarker discovery, renal transplant studies, testing prevention procedures, in addition to other clinical applications. The renal system in pigs is similar to that of the human based on anatomy, immunological response, organ homology (Lieberthal and Nigam 2000; (Lunney 2007; Piriou‐guzylack and Salmon 2008), and metabolic rate, all closer to humans when compared to rodents. Renal I/R in swine models has been performed in studies of I/R (Lieberthal and Nigam 2000; Simmon et al. 2008) and transplant models (Yang et al. 2012; Fonouni et al. 2011, 2012) to study new markers and therapeutic approaches (Hunter et al. 2012) using clamp occlusion and microdialysis with (Weld et al. 2008a; Hunter et al. 2012) and without (Weld et al. 2008b) contralateral nephrectomy.

Embolization particles, such as polyvinyl alcohol (PVA), have also been used to cause ischemia, but this method has partial vessel occlusion and has no control over reperfusion. One of the drawbacks of these methods is that particles must be of a specific size able to occlude the artery lumen. However, the arterial lumen varies among animals (Misra et al. 2006). Both models, the injection of PVA in the renal artery and the clamp model, did not have differences in relation to histology (Flacke et al. 2012). Furthermore, a study using constant injection of PVA (every 30 min for 2 h) showed heterogeneous results regarding the ischemic response (Keller et al. 2009) and the need to use an invasive method to evaluate blood flow.

The swine model allows us to use the catheter‐based occlusion, which is not possible in rodents. These catheters have been used because they are less invasive than clamp placement (Simon et al. 2011) enabling physiological responses only resulting from ischemia/reperfusion renal, without the confounding factor of the surgical aggression. A catheter‐based renal I/R was developed in pigs by introducing a balloon‐catheter into the abdominal aortic artery to occlude both renal arteries. This bilateral ischemia had higher renal injury and glomerular damage (dilatation of Bowman's space, swelling of Bowman's capsular cell (Matějková et al. 2013) and glomerular tubularization) (Simon et al. 2011) than our catheter‐based model.

Several studies have described the unilateral I/R model with nephrectomy (Susa et al. 2009; Flacke et al. 2012; Hunter et al. 2012) or bilateral I/R (Bhalodia et al. 2009; Matějková et al. 2013). Here, the unilateral model was chosen without unilateral nephrectomy due to a milder and minimally invasive aggression to identify physiological and biochemical changes earlier than elevation of serum creatinine. However, our model showed more histological injury than the ones previously described. Authors suggested that mild injury occurs by compensatory efforts of a solitary kidney or by pneumoperitoneal preischemia conditioning (Weld et al. 2008a). In our model, the contralateral kidney may be used as an individual control for each animal due to the absence of NTA. Contralateral kidneys have also been used as a control in other studies (Sabbagh et al. 2011; Damianovich et al. 2006).

In both animals and humans, AKI is characterized by the measurement of serum creatinine levels. Values equal to or higher than 150% from baseline are considered positive for AKI (Mehta et al. 2007). Our findings showed that after ischemia, only two animals would be classified as AKI (one of them in only one measurement), despite the histological data showing injury in all animals (with ATN and white and hemorrhagic necrosis). Nonetheless, levels of creatinine were significantly higher than baseline levels. This result did not correlate with Weld et al. (2008b) who did not show changes in serum creatinine 5 h after unilateral ischemia (90‐ and 120‐min occlusion). Also, serum creatinine did not increase even 24 h after occlusion of 120 and 150 min, but it only increased in occlusions greater than 180 min (Weld et al. 2008a). This lack of increase in serum creatinine occurred probably because the levels of baseline serum creatinine generated by surgery or nephrectomy were already high (~1.5 mg/dL) or because the assessment of serum creatinine was conducted earlier than 6 h after reperfusion in the first study, since in our model the increase was observed after this period. Finally, as expected, our model was characterized by a later increase in creatinine compared to models that occluded the two kidneys through the aorta for 90 min (Simon et al. 2011) and 120 min (Matějková et al. 2013), showing an increased level of serum creatinine after 2 and 4 h, respectively.

As the diagnosis of AKI using serum creatinine is known to have several limitations, many different biomarkers have been studied (Simsek et al. 2013). NGAL is widely used in studies (Mishra et al. 2005; Boldt et al. 2009) as an earlier marker than creatinine in AKI (Clerico et al. 2012). Increase in urinary (Silberstein et al. 2013; Hunter et al. 2012) and serum concentrations of NGAL (Silberstein et al. 2013; Jochmans et al. 2011; Matějková et al. 2013) were found in previous renal I/R swine models. Silberstein and cols showed an increase in serum NGAL after ischemia, although there was no difference between the unilateral I/R by a clamp hilar group and control (Silberstein et al. 2013). Other studies showed an increase 48 h after renal transplantation (Jochmans et al. 2011) and after 8 h of reperfusion of bilateral renal I/R of 120 min (Matějková et al. 2013).

In our model, serum NGAL showed a significant difference between ischemia and reperfusion. Several studies have suggested that ischemia stimulates an NGAL increase in tissue and serum, but the mechanism for that remains unclear (Chakraborty et al. 2012). In the present model, the use of serum NGAL was similar, not better, to using serum creatinine as a marker of AKI.

Another important process in characterization of I/R, and one of the factors responsible for NTA, is the increase in reactivity oxygen and nitrogen species. These species trigger lipid peroxidation, leading to destruction of cell membranes, DNA breakage, oxidation, and nitration of proteins (Li and Jackson 2002). The increase in nitrated proteins was demonstrated in urine and serum during ischemia and reperfusion, respectively, in this model.

The decline of NGAL, as well as in the concentration of serum‐nitrated proteins during ischemia and early reperfusion, was observed in all animals. This may have occurred due to an increase in urinary proteins in this period. The increase in protein excretion could originate from glomerular or tubular injury. I/R may lead to glomerular capillary permeability (Rippe et al. 2006), alterations in endothelial glycocalyx (Platts et al. 2003), and reduced expression and activity of the ionic transporter in the proximal tubule (Alejandro et al. 1995; Di Sole et al. 2011). In relation to the pattern of urinary protein in SDS‐PAGE, we found proteins with high molecular weight being excreted in the urine during ischemia and early reperfusion, similar to that described in transplanted individuals (Stefanidis et al. 1996; Artz et al. 2014). This suggests that there was slight structural glomerular injury in this period, confirmed by the excretion of nephrin (data not shown). However, these proteins are not maintained even after 18 h. Excretion of these proteins may have been generated by increased glomerular pressure during kidney ischemia with increased flow on the contralateral, followed by increased flow in the ischemic kidney after reperfusion. In the beginning of ischemia, podocytes use to undergo flattening and spreading (Racusen et al. 1984). These changes, associated with decreased heparin sulfate in the glomerular basement membrane during ischemia, may lead to reduced selectivity for load and size (increasing the glomerular pores). The longer the duration of ischemia, the greater will be the damage (Rippe et al. 2006).

However, the major injury generated by renal I/R occurs in the proximal tubule (Ponticelli and Graziani 2012). Renal ischemia reduces ATP, mainly affecting reabsorption through ATPases. This energetic reduction leads to a loss of brush border membrane and polarity with a reduction in Na^+^K^+^ATPase activity, which provides cotransport of other molecules like potassium, bicarbonate, and glucose against the electrochemical gradient. Therefore, the loss of these carriers or their activity leads to an increase in sodium, chloride, bicarbonate, glucose, and potassium excretion as shown (Kim et al. 1995; Spiegel et al. 1989). Increase in urinary glucose also characterizes tubular injury. All filtered glucose is reabsorbed, 90% in the proximal tubule. Previous works with ischemic insults showed glycosuria by reduction and impairment in tubular glucose transporters (Runembert et al. 2004). Our model showed all these described changes.

Our study has potentials limitations. In our model, the initial time used as a control (preischemia) was performed just prior to the occlusion of the renal artery, after the start of surgery, but we have not controlled for systemic changes following only the surgical procedure. The use of a sham control group could provide time‐series information on the surgical wound consequences. We have only used one occlusion time for deriving ischemic biomarkers (120 min) and one should acknowledge that our results could differ if shorter ischemia times were used. Finally, the urine collected from both kidneys and the blood from vena cava do not reflect the biochemical changes only from the ischemic kidney. However, the use of metabolite mixture from both kidneys was chosen to mimic the conditions of urine collection in clinical practice, where the metabolites used as a diagnostic tool represent both kidneys.

With this model, we identified important physiological responses, such as increased oxidative stress, cell death, increase in urinary excretion of proteins and ions, and also a deficit in glucose reabsorption by the kidney. Swine model more accurately reflects the changes occurring in humans, not only because of the anatomical similarities but also due to a metabolic rate closer to humans when compared to rodents. Therefore, future studies of metabolomics and proteomics with this model can characterize the pathophysiology of the disease and provide novel biomarkers for diagnosis, prognosis, and monitoring of therapeutic response, necessary for renal I/R and consequently AKI.

## Conclusion

Altogether, these data show that the renal ischemia/reperfusion model could be performed by interventional radiology techniques using a percutaneous approach in a suitable large mammalian model. This model will allow the development of studies to explore new early kidney ischemic biomarkers and initial pathophysiology response.

## Acknowledgments

We acknowledge the Nuclear Magnetic Resonance facility at Brazilian Biosciences National Laboratory (LNBio), CNPEM, Campinas, Brazil for the use of the 600 MHz Agilent Nuclear Magnetic Resonance Spectrometer for support data collection.

## Conflict of Interest

The authors declare that they have no conflict of interest
